# Systematic review and meta-analysis on the efficacy and safety of rimegepant for migraine

**DOI:** 10.3389/fneur.2026.1828779

**Published:** 2026-05-01

**Authors:** Tong An, Xiwen Duan, Yixuan Liu, Jingqi Chuang, Jiamei Liu, Wenbo Shi, Zhixiang Liu

**Affiliations:** College of Bioscience and Biotechnology, Shenyang Agricultural University, Shenyang, China

**Keywords:** freedom from MBS, meta-analysis, migraine, pain freedom, rimegepant

## Abstract

**Background:**

Migraine is a prevalent neurological condition, affecting approximately 14% of the population, and is a leading cause of disability. Conventional treatments may have limited efficacy and tolerability limitations in clinical practice. Rimegepant is approved for the dual indications of managing acute migraine episodes and preventing migraine attacks. Therefore, we sought to evaluate the efficacy and safety of rimegepant in migraine treatment.

**Method:**

A systematic search of public databases from their inception until February 2025 was conducted to identify randomized controlled trials for evaluating the efficacy of rimegepant in migraine treatment. The overall estimate was derived using the 95% confidence interval (CI) and risk ratio (RR) in a random-effects model. GRADEprofiler was employed to assess the quality of evidence. The Cochrane Collaboration was utilized to evaluate the risk of bias.

**Results:**

Our investigation reviewed 5 studies, which included 2,715 individuals treated with rimegepant and 2,850 control participants. Patients treated with rimegepant had statistically significant pain relief (RR = 1.34; 95% CI 1.27–1.41; *p* < 0.05; *I*^2^ = 0%) and freedom from Most Bothersome Symptom (MBS) 2 h after treatment (RR = 1.38; 95% CI 1.28–1.49; *p* < 0.05; *I*^2^ = 0%). However, the initial therapeutic success with rimegepant does not correlate with a sustained lack of pain relapse from 2 h to 48 h (RR = 1.16; 95% CI 0.99–1.37; *p* > 0.05; *I*^2^ = 0%). There was no statistically significant difference in adverse events observed between the treatment group and the control group (RR = 1.12; 95% CI 0.97–1.28; *p* > 0.05; *I*^2^ = 0%).

**Conclusion:**

Our findings suggest that rimegepant may be effective and generally well tolerated for acute migraine treatment. However, the drug did not demonstrate statistical significance for preventing pain relapse within 2–48 h following administration. Given the limitations in the quality and quantity of the included trials, the findings should be confirmed by multicenter, large-sample, randomized controlled trials.

**Systematic review registration:**

https://www.crd.york.ac.uk/PROSPERO/view/CRD420251180156, identifier: CRD420251180156.

## 1 Introduction

Migraines are a prevalent and debilitating neurological condition that manifests as recurring episodes of moderate to severe unilateral pulsatile headache, often accompanied by symptoms such as nausea, vomiting, photophobia, and phonophobia (1, 2). The 2021 Global Burden of Disease (GBD) study reveals that migraines affect about 1.16 billion people worldwide, with a global prevalence of approximately 14% (3). It frequently occurs in the age group with the highest personal productivity and is usually two to three times more common in women than in males. However, it is frequently overlooked and undertreated (2, 4). Traditional treatment strategies are divided into two categories: one is acute treatment, intended to alleviate symptoms during attacks, and the other is preventive therapy, designed to decrease the incidence and severity of migraine episodes (5). However, many of these conventional treatments may be associated with suboptimal efficacy and tolerability issues in clinical practice (4), thus driving the focus of research toward discovering novel therapeutic targets.

In both the central and peripheral nervous systems, the neuropeptide calcitonin gene-related peptide (CGRP) is extensively distributed. During a migraine attack, the released neuropeptide is considered to exert a key role in regulating nociceptive signal transmission and vasodilatation in the trigeminal vascular system (6). Rimegepant (Nurtec® ODT) acts as a CGRP receptor small molecule antagonist that works by blocking the CGRP receptor. As a member of the “gepants” class, it is used for the acute and preventive treatment of migraine (7). By competitively inhibiting CGRP receptors, it prevents the downstream effects of CGRP without inducing the vasoconstriction associated with tricyclic drugs (8, 9). This mechanism of action provides a promising alternative therapy for patients who have contraindications to, are unresponsive to, or are unable to tolerate triptans (7). As the first and only drug approved for both the acute treatment of migraine attacks (with or without aura) and the preventative treatment of episodic migraines in adults, rimegepant has become a vital therapeutic regimen for migraines. This dual therapeutic effect represents an innovative aspect of oral rimegepant drugs (10).

The effectiveness and safety of rimegepant have been examined in several clinical trials. Although in the large majority of studies, for both acute migraine treatment, rimegepant has demonstrated superior efficacy to placebo, some inconsistent findings remain. We performed a comprehensive quantitative evaluation of the safety and effectiveness of rimegepant in treating migraines in current randomized controlled trials. Consequently, this meta-analysis sought to offer an accurate, reliable, and detailed insight into the role of rimegepant in the management of migraines.

## 2 Methods

### 2.1 Study design

This study followed the guidelines outlined in the PRISMA Statement and the Cochrane Handbook (CH) for Systematic Reviews of Interventions (11). Additionally, the study has been registered (CRD420251180156) in the PROSPERO database.

### 2.2 PICO framework

The following inclusion criteria were set: (1) Study population: Adult patients who have a minimum history of 1 year with migraine and meet the International Classification of Headache Disorders, Third Edition (beta version) criteria, with or without aura. Furthermore, patients needed to be able to differentiate between migraine attacks and other types of headaches. (2) Intervention: 75 mg oral rimegepant tablet; (3) Comparison group: placebo; (4) The outcome measures included, complete freedom from pain, freedom from MBS and photophobia/phonophobia, pain relief, freedom from nausea (within 2 h), sustained pain freedom/relief (2–24 h and 2–48 h), no pain relapse (2–48 h), normal functioning at 2 h, and no rescue medication within 24 h. Safety outcomes encompassed any adverse events, risk of nausea and dizziness, risk of upper respiratory tract infection and urinary tract infection, severe adverse events, and adverse events associated with treatment; (5) Full-text, peer-reviewed randomized controlled trials (RCTs) with a parallel-group design in the study design. (6) Time frame: No restrictions on the publication date, but limitation of search to studies published in English.

### 2.3 Eligibility criteria

The following standards were used to choose which studies to include in this review: (1) research on adult patients with migraines; (2) randomized controlled trials (RCTs); (3) research comparing rimegepant (75 mg dose) with a placebo for the acute treatment of migraine; (4) research with at least one result.

### 2.4 Exclusion criteria

The following standards were used to choose which studies to exclude in this review: (1) research not using rimegepant as the principal pharmaceutical intervention for the acute treatment of migraine; (2) observational studies, conference abstracts, correspondences to the editor, reviews, and editorials; (3) research conducted without disclosing any pre-established effectiveness or safety results.

### 2.5 Search strategy

PubMed, Embase, and Cochrane Library databases were searched using text, keywords, and medical subject headings (MeSH) such as “migraine” and “rimegepant” to identify relevant literature (from inception to February 2025). Additional papers were obtained by screening references to review eligible articles. For more comprehensive information, we also searched clinicaltrials.gov.

### 2.6 Study selection

A reference management program was used to import all of the detected records and check them for duplicates. Two investigators screened titles and abstracts of each article independently for relevance. They acquired and read each article’s complete text to determine whether it met the predetermined inclusion criteria (XD and YL). If there were any disagreements, JL, the third reviewer, settled them.

### 2.7 Data extraction

Utilizing a standardized data extraction sheet, reviewers XD and YL independently extracted data from the included studies. Discrepancies were addressed through reaching a consensus. Information gathered encompassed trial characteristics (study design, first author’s name, NCT number, intervention type and dosage, publication year, funding source), patient demographics (mean age, gender, population, BMI, monthly frequency of moderate or severe attacks), and migraine outcomes.

### 2.8 Quality assessment

The included research was evaluated with the CH by two investigators, respectively (XD and YL). Assessment included blinding of participants and personnel, selective reporting, allocation concealment, random sequence generation, blinding of outcome assessment, incomplete outcome data, and other biases (XD and YL). The quality of each outcome was estimated by researchers using the Grading of Recommendations Assessment, Development and Evaluation (GRADE) system.

### 2.9 Data analysis

RevMan 5.3 software was employed for meta-analysis. The overall estimate was computed using a random-effects model. The combined effects were assessed using risk ratios (RRs) and 95% confidence intervals (CIs). The *I*^2^ test and Cochran’s *Q*-test were used to evaluate heterogeneity. Meeting the criteria of a *p*-value < 0.10 or *I*^2^ > 50% defined significant heterogeneity. Employing a leave-one-out method, Stata 11.0 carried out a sensitivity analysis. The quality of the outcome was evaluated using the GRADE system. For the identification of publication bias, Begg’s test, Egger’s test, and funnel plot were performed. A two-sided *p*-value below 0.05 indicated statistical significance.

## 3 Results

### 3.1 Search result

**Figure 1:**
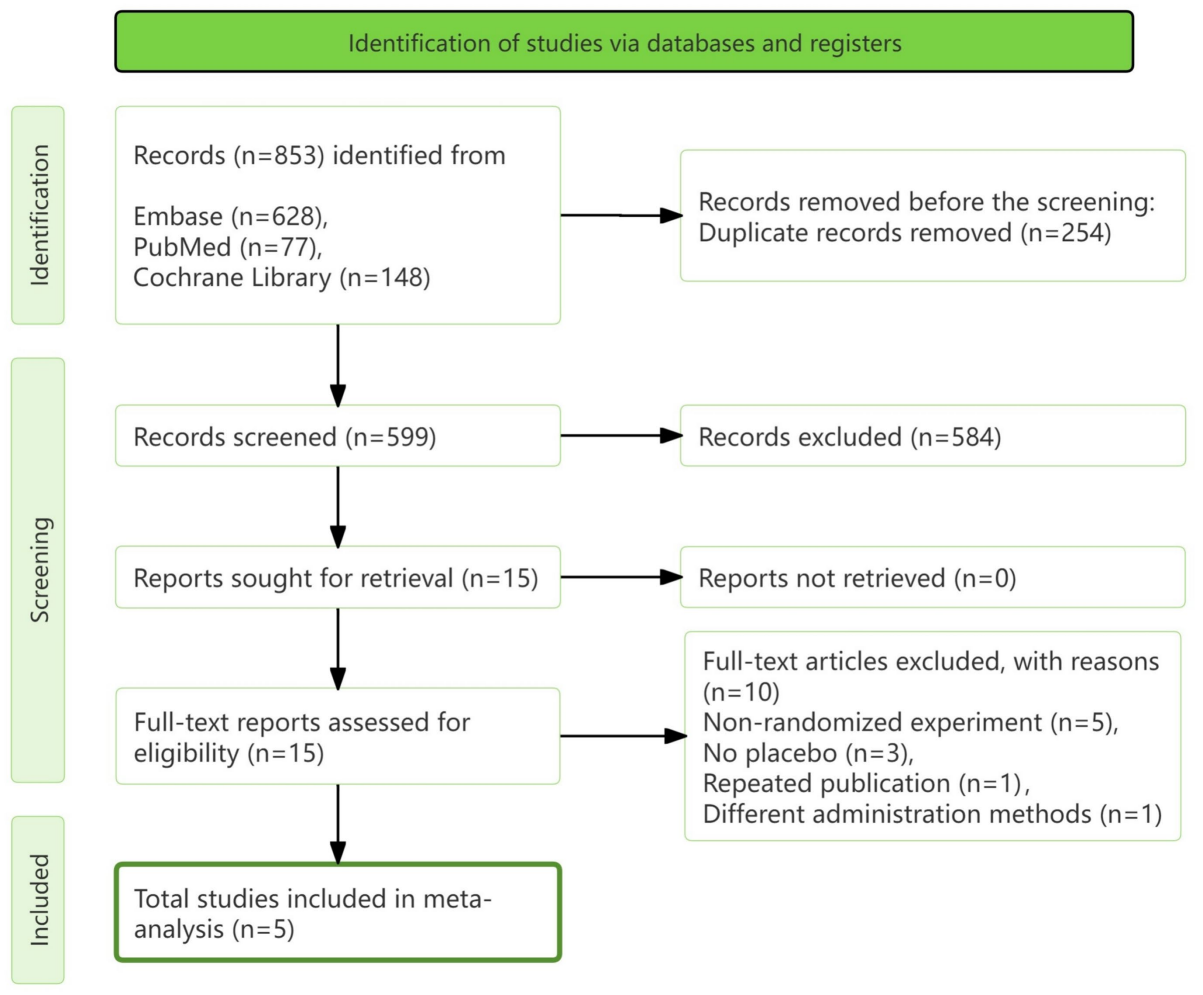
PRISMA diagram of study selection.

Initially, 853 articles were retrieved during the literature search. After excluding 254 duplicates and eliminating 584 that did not meet the inclusion criteria, 15 articles were available for full-text screening. Ultimately, 5 studies were included in our research (12–16). The selection process is outlined in detail in Figure 1.

### 3.2 Baseline characteristics

The publication dates of all included studies ranged from 2014 to 2024. All the study designs were double-blind RCTs. The analysis included a total of 5,565 participants, with 2,850 in the control group and 2,715 in the rimegepant group. The patients included in the study displayed moderate to severe migraines, with the majority being women (85%). Table 1 illustrates the patient characteristics and the corresponding trials.

**Table 1:** Characteristics of included trails.

Trial	NCT	Design	Participants, *n*	Mean age (years)	Gender (female %)	Country	BMI	Moderate or severe attacks per month, *n*	Therapeutic drugs (dose)	Source of funds
Lipton et al. (14)	NCT03235479	Double-blind RCT	582/580	41.9/41.3	F/M (85.5%)	United States	30.1/29.8	4.8/4.7	Rimegepant (75 mg)	Biohaven
Croop et al. (12)	NCT03461757	Double-blind RCT	732/734	40.3/40.0	F/M (85%)	United States	31.1/30.6	4.6/4.5	Rimegepant (75 mg)	Biohaven
Lipton et al. (20)	NCT03237845	Double-blind RCT	594/592	40.2/40.9	F/M (88.7%)	United States	31.0/31.8	4.5/4.6	Rimegepant (75 mg)	Biohaven
Yu et al. (16)	NCT04574362	Double-blind RCT	716/715	37.0/36.0	F/M (81%)	China/South Korea	22.5/22.7	3.3/3.3	Rimegepant (75 mg)	BioShin Limited
Marcus et al. (5)	NCT01430442	Double-blind RCT	91/229	38.5/37.9	F/M (87%)	United States	NA	3.91/4.00	Rimegepant (75 mg)	Bristol-Myers Squibb

NA, Not Available.

### 3.3 Assessment of bias risk and evidence quality

The studies included provided a clear explanation of how random sequence generation was conducted, primarily utilizing interactive online response systems. All studies used central allocation to achieve allocation concealment. Three of the five studies lacked sufficient information to assess the performance and detection risk (13–15). All five studies reported the number of loss to follow-up cases, and the analysis indicated that these loss to follow-up cases would not influence the results. Hence, the likelihood of incomplete outcome data in the five studies was deemed low. All studies have been registered on the ClinicalTrials.gov website and have been assigned National Clinical Trial (NCT) numbers. Selective reporting of these studies was judged to be low risk according to an evaluation of the trial protocol. Given that all studies were funded by corporations, every relevant study has been recognized as presenting unclear risks concerning other potential risks. The assessment of each study was summarized in Table 2.

**Table 2:** Summary of evidence calssification.

Outcome indicators	Studies	Design	Risk of bias	Inconsistency	Indirectness	Imprecision	Publication bias	Quality	Importance
Pain freedom at 2 h	4	RCT	Serious^a^	No serious	No serious	No serious	Serious^d^	Low	Critical
Freedom from MBS at 2 h	4	RCT	Serious^a^	No serious	No serious	No serious	Serious^d^	Low	Critical
Photophobia freedom at 2 h	4	RCT	Serious^a^	No serious	No serious	No serious	Serious^d^	Low	Important
Phonophobia freedom at 2 h	4	RCT	Serious^a^	No serious	No serious	No serious	Serious^d^	Low	Important
Pain relief at 2 h	5	RCT	Serious^a^	No serious	No serious	No serious	Serious^d^	Low	Important
Nausea freedom at 2 h	4	RCT	Serious^a^	No serious	No serious	No serious	Serious^d^	Low	Important
Sustained pain freedom, 2–24 h	5	RCT	Serious^a^	Serious^b^	No serious	No serious	Serious^d^	Low	Important
Sustained pain relief, 2–24 h	4	RCT	Serious^a^	No serious	No serious	No serious	Serious^d^	Low	Important
Sustained pain freedom, 2–48 h	5	RCT	Serious^a^	No serious	No serious	No serious	Serious^d^	Moderate	Important
Sustained pain relief, 2–48 h	3	RCT	Serious^a^	No serious	No serious	No serious	Serious^d^	Low	Important
No pain relapse, 2–48 h	3	RCT	Serious^a^	No serious	No serious	No serious	Serious^d^	Low	Important
Able to function normally at 2 h	4	RCT	Serious^a^	No serious	No serious	No serious	Serious^d^	Low	Not important
No rescue medication within 24 h	4	RCT	Serious^a^	No serious	No serious	No serious	Serious^d^	Low	Important
Any adverse events	4	RCT	Serious^a^	No serious	No serious	No serious	Serious^d^	Low	Important
Nausea	5	RCT	Serious^a^	No serious	No serious	No serious	Serious^d^	Low	Important
Dizziness	3	RCT	Serious^a^	No serious	No serious	Serious^c^	Serious^d^	Very low	Important
Upper respiratory tract infection	2	RCT	Serious^a^	No serious	No serious	No serious	Serious^d^	Low	Important
Urinary tract infection	3	RCT	Serious^a^	No serious	No serious	No serious	Serious^d^	Low	Important
Serious adverse event	4	RCT	Serious^a^	No serious	No serious	No serious	Serious^d^	Low	Critical
adverse events related to treatment	2	RCT	Serious^a^	No serious	No serious	No serious	Serious^d^	Low	Critical

a Every study included in the analysis had at least one risk of bias.

b There is moderate heterogeneity, *I*^2^ = 54%.

c The relatively small number of patients and observed events included in the study leads to too wide confidence interval.

d Reporting bias exists.

**Figure 2:**
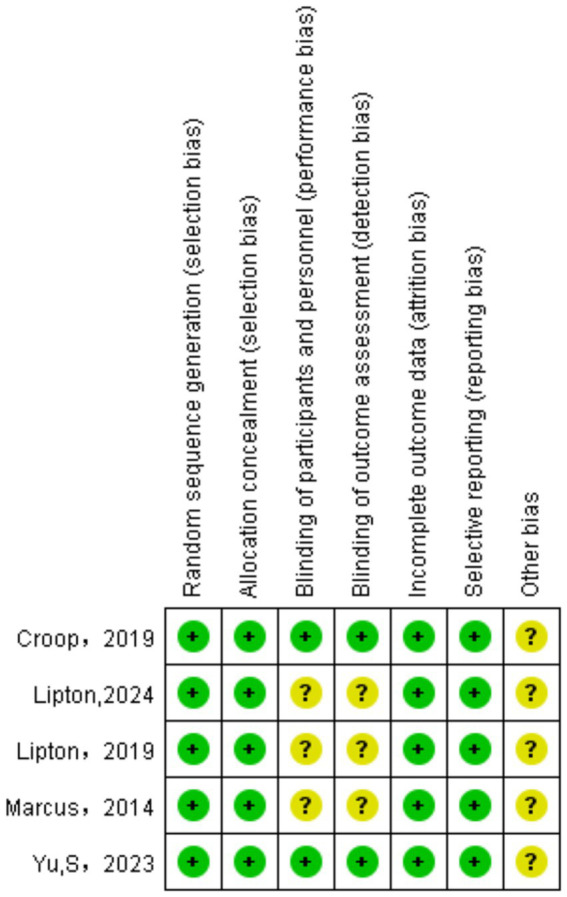
Assessment of bias risk for included studies.

The evidence classification was shown in Table 2, which was evaluated using GRADEprofiler. The quality of evidence on “sustained freedom from pain for 2–48 h” was moderate. The quality of evidence on “dizziness” was very low. The evidence quality for the remaining outcomes was deemed low (Figure 2).

### 3.4 Efficacy outcomes

#### 3.4.1 Freedom from pain at 2 h

**Figure 3:**
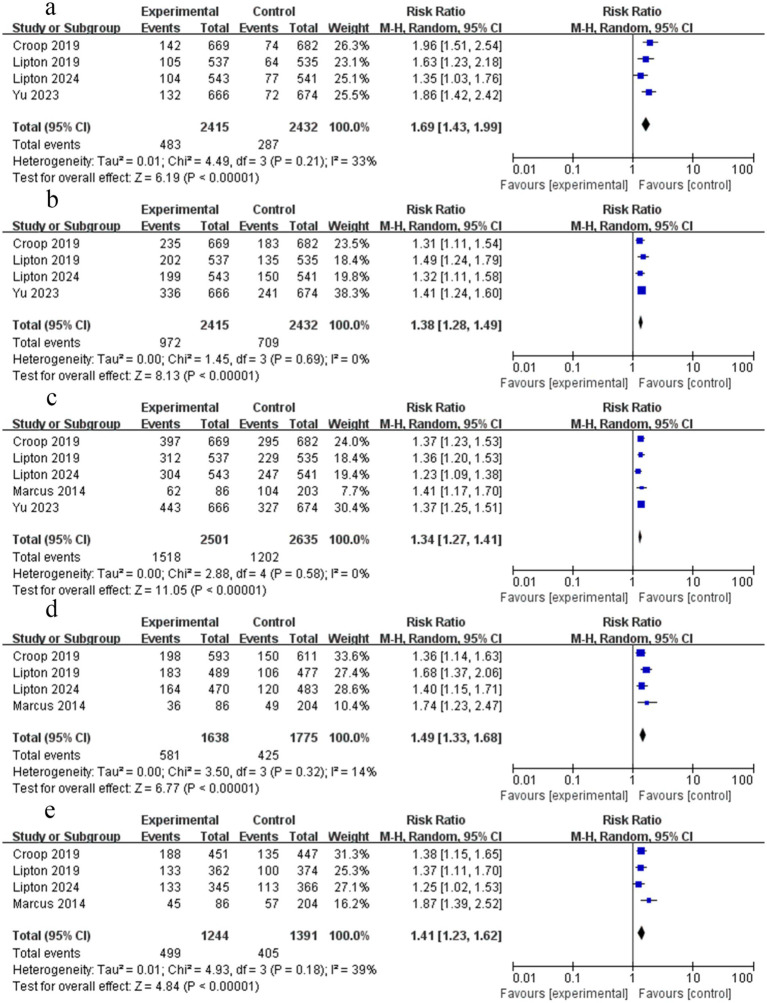
Forest plot of rimegepant efficacy on migraine. **(a)** Pain freedom at 2 h; **(b)** Freedom from MBS at 2 h; **(c)** Pain relief at 2 h; **(d)** Photophobia freedom at 2 h; **(e)** Phonophobia freedom at 2 h.

Four RCTs reported data on freedom from pain 2 h after the dose. The findings indicated that rimegepant therapy was associated with a higher likelihood of freedom from pain at 2 h (RR = 1.69; 95% CI 1.43–1.99; *p* < 0.05; *I*^2^ = 33%) (Figure 3a). The funnel plot appeared symmetrical and Begg’s test (*p* = 0.089) showed no publication bias, whereas Egger’s test (*p* = 0.038) yielded a conflicting result.

#### 3.4.2 Freedom from MBS at 2 h

Four RCTs provided data on the absence of MBS 2 h after rimegepant administration. The findings of the meta-analysis demonstrated that patients experienced freedom from MBS at 2 h (RR = 1.38; 95% CI 1.28–1.49; *p* < 0.05; *I*^2^ = 0%) following treatment with rimegepant (Figure 3b). No significant publication bias was observed based on the symmetric funnel plot, Begg’s test (*p* = 1.000), and Egger’s test (*p* = 0.805).

#### 3.4.3 Pain relief at 2 h

Five RCTs reported data on pain relief at 2 h post-dose. The findings of the meta-analysis indicated that rimegepant therapy provided pain relief within 2 h (RR = 1.34; 95% CI 1.27–1.41; *p* < 0.05; *I*^2^ = 0%) (Figure 3c). Although visual inspection of the funnel plot suggested potential asymmetry, no significant publication bias was identified by Begg’s test (*p* = 1.000) and Egger’s test (*p* = 0.402).

#### 3.4.4 Freedom from photophobia at 2 h

Four RCTs reported data on freedom from photophobia at 2 h. The findings of the meta-analysis demonstrated that rimegepant therapy provided freedom from photophobia at 2 h (RR = 1.49; 95% CI 1.33–1.68; *p* < 0.05; *I*^2^ = 14%) (Figure 3d). No significant publication bias was observed based on the symmetric funnel plot, Begg’s test (*p* = 0.734), and Egger’s test (*p* = 0.288).

#### 3.4.5 Freedom from phonophobia at 2 h

Four RCTs reported data on freedom from phonophobia at 2 h. Rimegepant therapy was associated with a significantly higher likelihood of freedom from phonophobia at 2 h (RR = 1.41; 95% CI 1.23–1.62; *p* < 0.05; *I*^2^ = 39%) (Figure 3e). Moderate heterogeneity was present (*I*^2^ = 39%). While the funnel plot appeared asymmetrical, Begg’s test (*p* = 0.734) and Egger’s test (*p* = 0.105) showed no significant publication bias.

#### 3.4.6 Relief from nausea at 2 h

**Figure 4:**
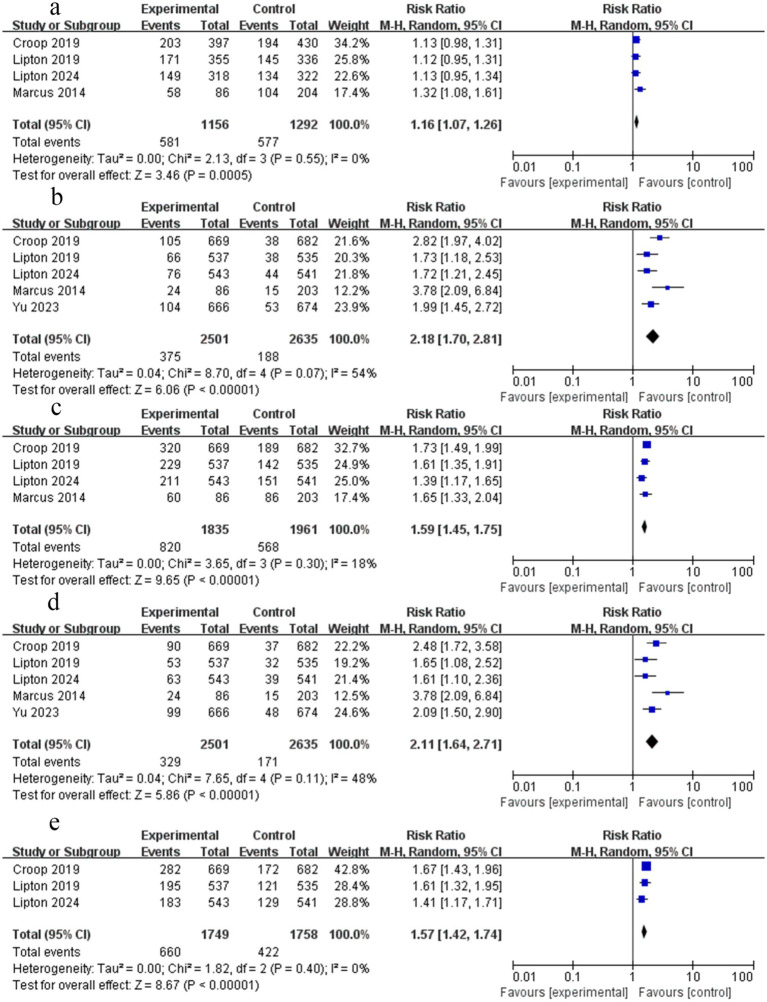
Forest plot comparing the rimegepant efficacy of migraine. **(a)** Nausea freedom at 2 h; **(b)** Sustained pain freedom, 2–24 h; **(c)** Sustained pain relief, 2–24 h; **(d)** Sustained pain freedom, 2–48 h; **(e)** Sustained pain relief, 2–48 h.

Four RCTs reported data on relief from nausea at 2 h. The findings of the meta-analysis demonstrated that rimegepant therapy provided relief from nausea at 2 h (RR = 1.16; 95% CI 1.07–1.26; *p* < 0.05; *I*^2^ = 0%) (Figure 4a). No significant publication bias was detected by Begg’s test (*p* = 0.734), although the asymmetric funnel plot and significant Egger’s test (*p* = 0.030) yielded inconsistent results.

#### 3.4.7 Sustained pain freedom from 2 to 24 h

Five RCTs reported data on sustained pain freedom from 2 h to 24 h. The findings of the meta-analysis demonstrated that rimegepant was associated with a significantly higher likelihood of sustained pain freedom from 2 h to 24 h (RR = 2.18; 95% CI 1.70–2.81; *p* < 0.05; *I*^2^ = 54%) (Figure 4b). Moderate heterogeneity was present (*I*^2^ = 54%). While the funnel plot appeared asymmetrical, Begg’s test (*p* = 1.000) and Egger’s test (*p* = 0.331) showed no significant publication bias.

#### 3.4.8 Sustained pain relief from 2 to 24 h

Four RCTs reported data on sustained pain relief from 2 h to 24 h. The findings of the meta-analysis demonstrated that rimegepant was associated with a significantly higher sustained pain relief from 2 h to 24 h (RR = 1.59; 95% CI 1.45–1.75; *p* < 0.05; *I*^2^ = 18%) (Figure 4c). No significant publication bias was observed based on the symmetric funnel plot, Begg’s test (*p* = 1.000), and Egger’s test (*p* = 0.609).

#### 3.4.9 Sustained pain freedom from 2 to 48 h

Five RCTs reported data on sustained pain freedom from 2 h to 48 h. The findings of the meta-analysis demonstrated that rimegepant was associated with a significantly higher likelihood of sustained pain freedom from 2 h to 48 h (RR = 2.11; 95% CI 1.64–2.71; *p* < 0.05; *I*^2^ = 48%) (Figure 4d). Moderate heterogeneity was present (*I*^2^ = 48%). While the funnel plot appeared asymmetrical, Begg’s test (*p* = 1.000) and Egger’s test (*p* = 0.308) showed no significant publication bias.

#### 3.4.10 Sustained pain relief from 2 to 48 h

Three RCTs reported data on sustained pain relief from 2 h to 48 h. The findings of the meta-analysis demonstrated that rimegepant was associated with a significantly higher likelihood of sustained pain relief from 2 h to 48 h (RR = 1.57; 95% CI 1.42–1.74; *p* < 0.05; *I*^2^ = 0%) (Figure 4e). No significant publication bias was observed based on the symmetric funnel plot, Begg’s test (*p* = 0.296), and Egger’s test (*p* = 0.335).

#### 3.4.11 No pain relapse from 2 to 48 h

**Figure 5:**
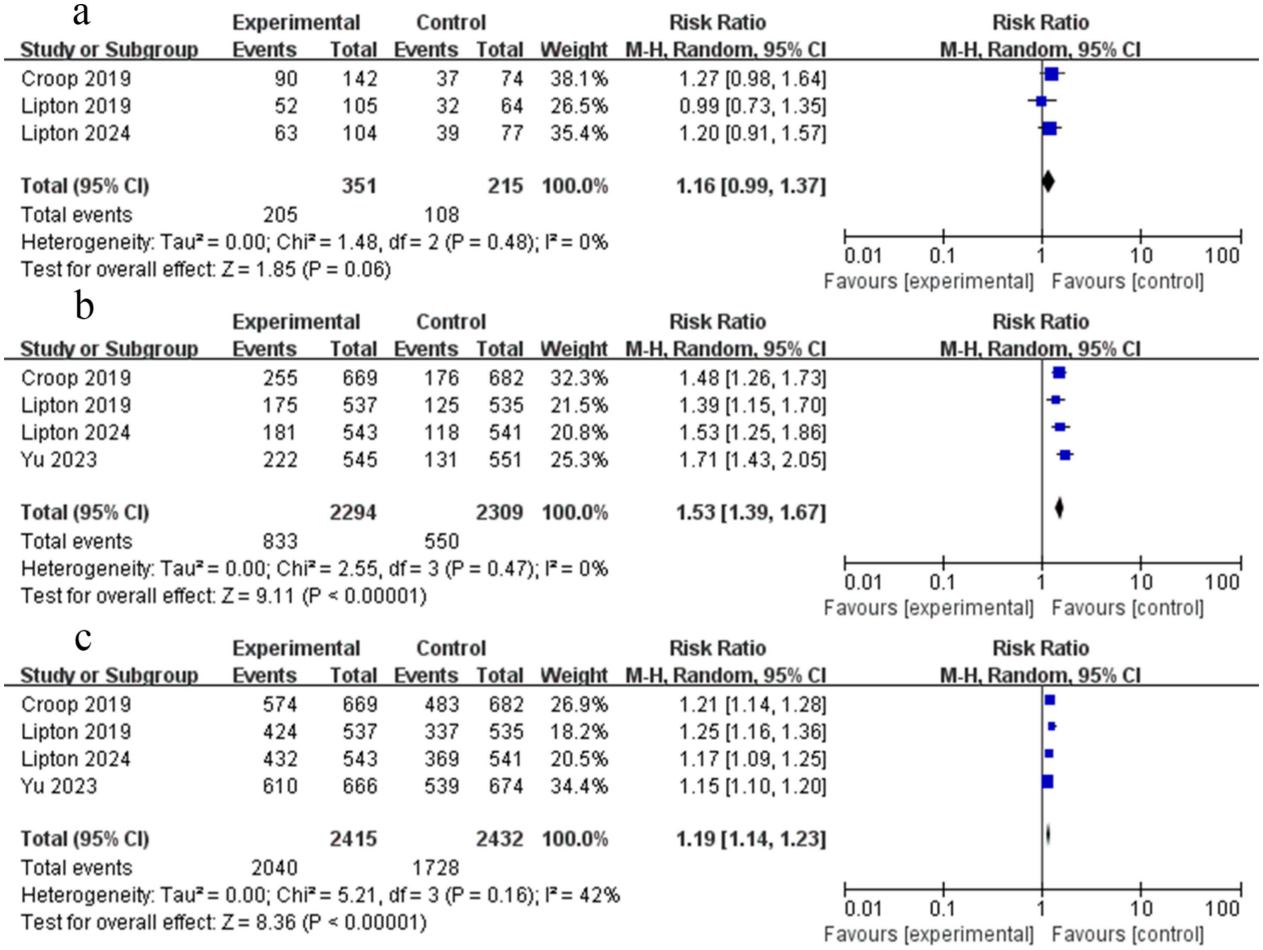
Forest plot of migraine outcomes between rimegepant and placebo: **(a)** No pain relapse, 2–48 h; **(b)** Able to function normally at 2 h; **(c)** No rescue medication within 24 h.

Three RCTs reported data on the absence of pain relapse from 2 h to 48 h. The meta-analysis demonstrated that rimegepant was not associated with a significant reduction in pain relapse from 2 h to 48 h (RR = 1.16; 95% CI 0.99–1.37; *p* > 0.05; *I*^2^ = 0%) (Figure 5a). No significant publication bias was observed based on the symmetric funnel plot, Begg’s test (*p* = 0.296), and Egger’s test (*p* = 0.539).

#### 3.4.12 Ability to function normally at 2 h

Four RCTs reported data on sustained ability to function normally at 2 h. The meta-analysis demonstrated that rimegepant was associated with a significantly higher likelihood of normal functional ability at 2 h (RR = 1.53; 95% CI 1.39–1.67; *p* < 0.05; *I*^2^ = 0%) (Figure 5b). No significant publication bias was observed based on the symmetric funnel plot, Begg’s test (*p* = 0.734), and Egger’s test (*p* = 0.579).

#### 3.4.13 No rescue medication within 24 h

Four RCTs reported data on the proportion of patients not using rescue medication within 24 h. The meta-analysis demonstrated that rimegepant was associated with a significantly higher likelihood of avoiding rescue medication within 24 h (RR = 1.19; 95% CI 1.14–1.23; *p* < 0.05; *I*^2^ = 42%) (Figure 5c). No significant publication bias was observed based on the symmetric funnel plot, Begg’s test (*p* = 0.734), and Egger’s test (*p* = 0.625).

### 3.5 Safety outcomes

#### 3.5.1 Documentation of any adverse events

**Figure 6:**
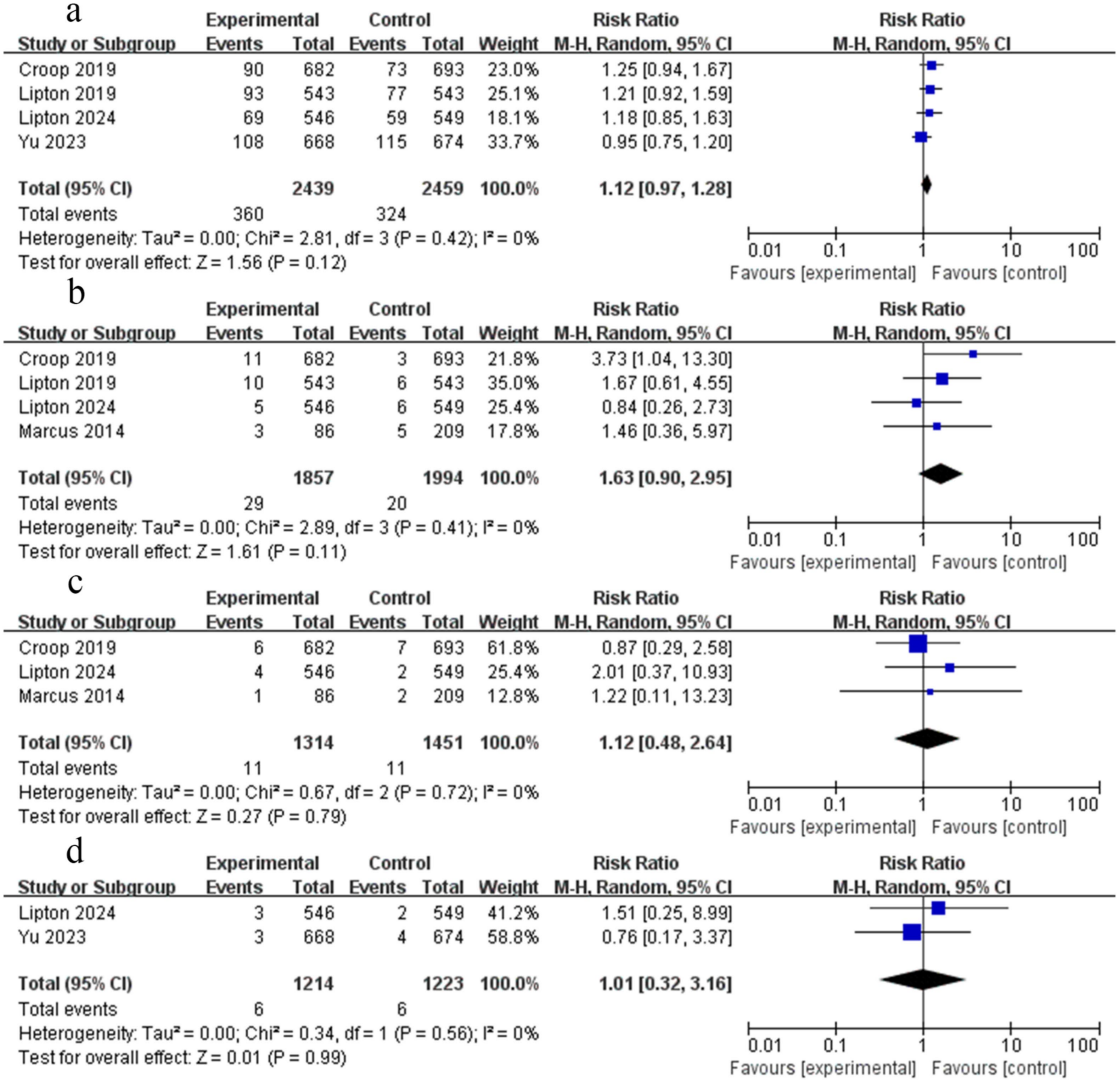
Forest plot of rimegepant safety on migraine. **(a)** Documentation of any adverse events; **(b)** the risk of nausea; **(c)** the risk of dizziness; **(d)** the risk of upper respiratory tract infection.

Four studies analyzed any adverse events of patients treated with rimegepant. In our analysis, there was no statistically significant difference between rimegepant therapy and placebo concerning any adverse events (RR = 1.12; 95% CI 0.97–1.28; *p* > 0.05; *I*^2^ = 0%) (Figure 6a). Although the funnel plot appeared asymmetrical, Begg’s test (*p* = 1.000), and Egger’s test (*p* = 0.719) did not reveal significant publication bias.

#### 3.5.2 The risk of nausea

Five studies analyzed the risk of nausea in patients treated with rimegepant. In our analysis, there was no statistically significant difference between rimegepant therapy and placebo concerning the risk of nausea (RR = 1.16; 95% CI 0.53–2.57; *p* > 0.05; *I*^2^ = 60%). Moderate heterogeneity was found (*I*^2^ = 60%), which was also suggested by an asymmetrical funnel plot. We therefore performed sensitivity analyses. After excluding the suspicious study from our analysis, the findings indicated that this study (17) was the source of the heterogeneity. The results of the remaining four RCTs were unchanged (RR = 1.63; 95% CI 0.90–2.95; *p* > 0.05; *I*^2^ = 0%) (Figure 6b). No significant publication bias was observed based on the symmetric funnel plot, Begg’s test (*p* = 0.734), and Egger’s test (*p* = 0.275).

#### 3.5.3 The risk of dizziness

Three studies analyzed the risk of dizziness in patients treated with rimegepant therapy. In our analysis, there was no statistically significant difference between rimegepant therapy and placebo concerning the risk of dizziness (RR = 1.12; 95% CI 0.48–2.64; *p* > 0.05; *I*^2^ = 0%) (Figure 6c). No significant publication bias was observed based on the symmetric funnel plot, Begg’s test (*p* = 1.000), and Egger’s test (*p* = 0.976).

#### 3.5.4 The risk of upper respiratory tract infection

Two studies analyzed the risk of upper respiratory tract infection in patients treated with rimegepant therapy. In our analysis, there was no statistically significant difference between rimegepant therapy and placebo concerning the risk of upper respiratory tract infection (RR = 1.01; 95% CI 0.32–3.16; *p* > 0.05; *I*^2^ = 0%) (Figure 6d). No significant publication bias was observed based on the symmetric funnel plot and Begg’s test (*p* = 1.000).

#### 3.5.5 The risk of urinary tract infection

**Figure 7:**
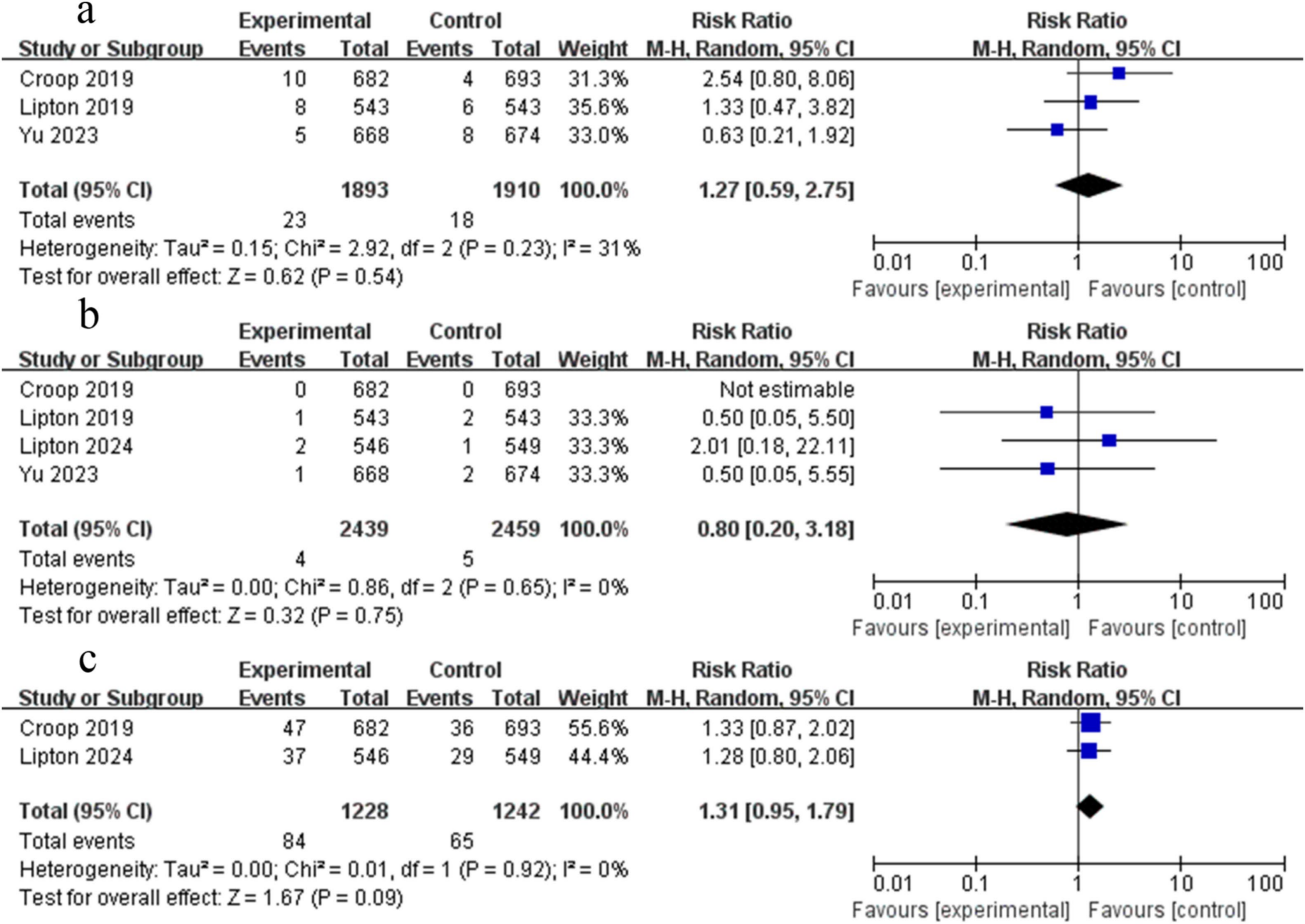
Forest plot comparing the rimegepant safety of migraine: **(a)** The risk of urinary tract infection; **(b)** Serious adverse events; **(c)** Adverse events related to treatment.

Three studies analyzed the risk of urinary tract infection in patients treated with rimegepant therapy. In our analysis, there was no statistically significant difference between rimegepant therapy and placebo concerning the risk of urinary tract infection (RR = 1.27; 95% CI 0.59–2.75; *p* > 0.05; *I*^2^ = 31%) (Figure 7a). No significant publication bias was detected based on the symmetric funnel plot, Begg’s test (*p* = 0.296), and Egger’s test (*p* = 0.083).

#### 3.5.6 Severe adverse events

Four studies analyzed the occurrence of severe adverse events in patients treated with rimegepant therapy. Data from one study were recorded as 0 and excluded from the statistical analysis (12). In our analysis, there was no statistically significant difference between rimegepant therapy and placebo concerning the occurrence of severe adverse events (RR = 0.80; 95% CI 0.20–3.18; p > 0.05; *I*^2^ = 0%) (Figure 7b). No significant publication bias was observed based on the symmetric funnel plot, Begg’s test (*p* = 0.296); however, Egger’s test indicated significant publication bias (*p* = 0.002).

#### 3.5.7 Adverse events related to treatment

Two studies analyzed the adverse events related to the rimegepant treatment in patients. In our analysis, there was no statistically significant difference between rimegepant therapy and placebo concerning adverse events related to the treatment (RR = 1.31; 95% CI 0.95–1.79; *p* > 0.05; *I*^2^ = 0%) (Figure 7c). No significant publication bias was observed based on the symmetric funnel plot and Begg’s test (*p* = 1.000).

## 4 Discussion

Migraines have been increasingly recognized as a major public health problem, yet patient satisfaction with available migraine treatments remains suboptimal. Clinical studies have focused on the therapeutic potential of agents targeting the CGRP pathway (12). Notably, the 2024 consensus statement from the American Headache Society has designated CGRP-targeted therapies as first-line options for migraine prevention, recommending their use without requiring an initial trial of conventional preventive agents (17, 18). Currently, approved CGRP-targeted therapeutics for migraines are primarily categorized into two classes: small-molecule CGRP receptor antagonists, including rimegepant, and CGRP/CGRP receptor monoclonal antibodies (19–21). While the latter are predominantly indicated for prophylactic use, the majority of gepants are restricted to acute migraine management. Rimegepant is approved for both the acute treatment and prevention of episodic migraines (EM). Rimegepant’s dual indication sets it apart from other agents in the same class like ubrogepant and zavegepant (10). The core advantages of CGRP-targeted drugs lie in their high specificity and favorable tolerability. CGRP-targeted drugs exhibit favorable efficacy and safety profiles compared with conventional preventive medications, and they have no cardiovascular contraindications, enabling a broader applicable population (22). However, studies have indicated that over 40% of patients show no response to CGRP-blocking agents, which may be associated with the independent role of the pituitary adenylate cyclase-activating polypeptide pathway (23). Thus, the limitations associated with rimegepant therapy in the treatment of migraines remain to be fully clarified.

This study pooled data from five RCTs with a total of 5,565 participants to evaluate the efficacy and safety of rimegepant therapy in the treatment of migraines. Our findings suggest that rimegepant may be associated with improved pain freedom and relief of migraine-associated symptoms, including photophobia, phonophobia, and nausea. The drug’s failure to prevent pain recurrence between 2 and 48 h may be attributed to two key factors. First, rimegepant’s mode of action is limited to selective blockade of the CGRP pathway. However, pain relapse also involves the compensatory upregulation of other pronociceptive peptides, and non-CGRP-mediated pathophysiological mechanisms lie outside the therapeutic scope of rimegepant (24). Second, a discrepancy exists between the duration of drug efficacy and the occurrence of pain relapse. With a terminal half-life of approximately 11 h (25), rimegepant can maintain pain relief or complete freedom from pain for 2 to 48 h after a single dose. Furthermore, pain relapse represents the initiation of a new pathophysiological cascade rather than simply the waning of drug effects (26). As such, selective blockade of the CGRP pathway alone is insufficient to block the new nociceptive signals that lead to migraine recurrence.

Findings from a 2023 meta-analysis suggested that rimegepant was associated with favorable outcomes in migraine management (27). Although our findings are consistent with those of previous studies, it is crucial to recognize the distinctions between our study and the prior research. Firstly, our meta-analysis included 13 efficacy outcomes and 7 safety outcomes, whereas the earlier meta-analysis only included a subset of these. The expanded outcomes set in this study allows a more comprehensive assessment of rimegepant’s clinical profile. Secondly, we incorporated two additional RCTs (14, 16). Furthermore, we performed a risk of bias assessment for all included trials and evaluated the quality of evidence, steps that were not conducted in the earlier meta-analysis. Thirdly, our findings may provide clinical guidance for using rimegepant to treat migraines, particularly regarding the risk of pain recurrence from 2 h to 48 h. These observations were not reported in previous meta-analyses.

Despite this, our study had certain limitations. Firstly, all included trials evaluated rimegepant as an acute treatment. The safety of rimegepant for preventive or long-term use remains unclear. Secondly, all of the studies received funding from pharmaceutical companies, and the risk-of-bias evaluation revealed unclear risk of publication bias. The GRADE profiling further showed that the overall evidence quality supporting our conclusions was assessed as low. Thirdly, the precision of the pooled estimate was hindered by a relatively limited sample size. Therefore, the findings should be interpreted cautiously until confirmed by large-sample, multicenter, randomized controlled trials.

## 5 Conclusion

Our systematic review and meta-analysis suggests that rimegepant may be associated with improved pain freedom and relief of associated symptoms in acute migraine treatment. However, the drug did not demonstrate a statistically significant effect on pain recurrence within 2–48 h after administration. Further large-scale clinical trials are warranted to validate the efficacy and safety of rimegepant, particularly to account for potential confounders, including follow-up duration, dosing regimens, and patient characteristics.

## Data Availability

The original contributions presented in the study are included in the article/Supplementary material, further inquiries can be directed to the corresponding author.
