# Supplementary material for: Systematic review and meta-analysis on the efficacy and safety of rimegepant for migraine
Source: Front Neurol. 2026 May 1;17:1828779. doi: 10.3389/fneur.2026.1828779 (PMC13175806; doi:10.3389/fneur.2026.1828779)
Supplement: Supplementary file 1 [file Table_1.DOC]

**Supplementary material**

**Table of Contents**

Appendix 1: Search strategy and search yields

Appendix 2: Assessment of bias risk of efficacy outcomes

Appendix 3: Assessment of bias risk of safety outcomes

**Appendix 1: Search strategy and search yields**

| **Search number** | **Query** | **Results** |
| --- | --- | --- |
| **PubMed** | | |
| #1 | Migraine | 43760 |
| #2 | Migraines | 3752 |
| #3 | Headaches | 27353 |
| #4 | Headache | 94433 |
| #5 | Migrainosus | 160 |
| #6 | Hemicrania | 987 |
| #7 | Cephalgia | 351 |
| #8 | Cephalgias | 124 |
| #9 | [Cephalalgias](http://coch.ilibs.cn/advanced-search/mesh#0) | 402 |
| #10 | Ciliary Neuralgias | 0 |
| #11 | Ciliary Neuralgia | 4 |
| #12 | Hortons Syndrome | 30 |
| #13 | Horton's Syndrome | 30 |
| #14 | [Alice in Wonderland Syndrome](http://coch.ilibs.cn/advanced-search/mesh#0) | 174 |
| #15 | Rimegepant | 196 |
| #16 | Vydura | 1 |
| #17 | Nurtec | 17 |
| #18 | Nurtec ODT | 7 |
| #19 | groups | 2795602 |
| #20 | trial | 847135 |
| #21 | randomly | 445582 |
| #22 | placebo | 260137 |
| #23 | randomized | 728728 |
| #24 | drug therapy | 56148 |
| #25 | controlled clinical trial | 21148 |
| #26 | randomized controlled trial | 126508 |
| #27 | “Clinical Trial” | 224427 |
| #28 | “Clinical Trials as Topic” | 354 |
| #29 | OR/1-14 | 133943 |
| #30 | OR/15-18 | 196 |
| #31 | OR/19-28 | 3958226 |
| #32 | AND/29-31 | 77 |

| **Embase** | | |
| --- | --- | --- |
| #1 | Migraine | 94928 |
| #2 | Migraines | 6661 |
| #3 | Headaches | 45561 |
| #4 | Headache | 380961 |
| #5 | Migrainosus | 350 |
| #6 | Hemicrania | 1720 |
| #7 | Cephalgia | 595 |
| #8 | Cephalgias | 216 |
| #9 | [Cephalalgias](http://coch.ilibs.cn/advanced-search/mesh#0) | 589 |
| #10 | Ciliary Neuralgias | 2 |
| #11 | Ciliary Neuralgia | 19 |
| #12 | Hortons Syndrome | 161 |
| #13 | [Alice in Wonderland Syndrome](http://coch.ilibs.cn/advanced-search/mesh#0) | 314 |
| #14 | Rimegepant | 736 |
| #15 | Vydura | 8 |
| #16 | Nurtec | 49 |
| #17 | “Nurtec ODT” | 32 |
| #18 | groups | 3996738 |
| #19 | trial | 2810874 |
| #20 | randomly | 594350 |
| #21 | placebo | 558311 |
| #22 | randomized | 1536453 |
| #23 | drug therapy | 7115434 |
| #24 | controlled clinical trial | 1569234 |
| #25 | randomized controlled trial | 1229179 |
| #26 | “Clinical Trial” | 1917300 |
| #27 | “Clinical Trials as Topic” | 485 |
| #28 | OR/1-13 | 426266 |
| #29 | OR/14-17 | 737 |
| #30 | OR/18-27 | 11710093 |
| #31 | AND/28-30 | 628 |

| **Cochrane** | | |
| --- | --- | --- |
| #1 | Migraine | 9937 |
| #2 | Migraines | 684 |
| #3 | Headaches | 3679 |
| #4 | Headache | 36889 |
| #5 | Migrainosus | 13 |
| #6 | Hemicrania | 37 |
| #7 | Cephalgia | 18 |
| #8 | Cephalgias | 9 |
| #9 | [Cephalalgias](http://coch.ilibs.cn/advanced-search/mesh#0) | 20 |
| #10 | Ciliary Neuralgias | 0 |
| #11 | Ciliary Neuralgia | 1 |
| #12 | Hortons Syndrome | 1 |
| #13 | Horton's Syndrome | 5 |
| #14 | [Alice in Wonderland Syndrome](http://coch.ilibs.cn/advanced-search/mesh#0) | 0 |
| #15 | Rimegepant | 161 |
| #16 | Vydura | 6 |
| #17 | Nurtec | 5 |
| #18 | Nurtec ODT | 5 |
| #19 | groups | 627658 |
| #20 | trial | 1085925 |
| #21 | randomly | 330695 |
| #22 | placebo | 378317 |
| #23 | randomized | 1175044 |
| #24 | drug therapy | 544131 |
| #25 | controlled clinical trial | 644599 |
| #26 | randomized controlled trial | 760216 |
| #27 | “Clinical Trial” | 460953 |
| #28 | “Clinical Trials as Topic” | 41579 |
| #29 | OR/1-14 | 42046 |
| #30 | OR/15-18 | 161 |
| #31 | OR/19-28 | 1658122 |
| #32 | AND/29-31 | 148 |

**Appendix 2**: Assessment of bias risk of efficacy outcomes

2.1 Pain freedom at 2 hours

2.2 Freedom from MBS at 2 hours

2.3 Pain relief at 2 hours

2.4 Photophibia freedom at 2 hours

2.5 phonophobia freedom at 2 hours

2.6 Nausea freedom at 2 hours

2.7 Sustained pain freedom, 2-24 hours

2.8 Sustained pain relief, 2-24 hours

2.9 Sustained pain freedom, 2-48 hours

2.10 Sustained pain relief, 2-48 hours

2.11 No pain relapse, 2-48 hours

2.12 Able to functional normally at 2 hours

2.13 No rescue medication within 24 hours

**Appendix 3**: Assessment of bias risk of safety outcomes

3.1 Documentation of any adverse events

3.2 The risk of nausea

3.3 the risk of dizziness

3.4 The risk of upper respiratory tract infection

\

3.5 The risk of urinary tract infection

3.6 Serious adverse events

3.7 Adverse events related to treatment
